# Prebiotic-like Effects of Proanthocyanidin-Rich Aronia Extract Supplementation on Gut Microbiota Composition and Function in the Twin-M-SHIME^®^ Model

**DOI:** 10.3390/ph18060793

**Published:** 2025-05-25

**Authors:** Blanca Elizabeth Ruiz-Álvarez, Valentina Cattero, Yves Desjardins

**Affiliations:** 1 Institute of Nutrition and Functional Foods (INAF), Faculty of Agriculture and Food Sciences, Laval University, Quebec, QC G1V 0A6, Canada; blanca-elizabeth.ruiz-alvarez.1@ulaval.ca (B.E.R.-Á.);; 2 Centre Nutrition, Santé et Société (NUTRISS), INAF Laval University, Quebec, QC G1V 0A6, Canada; 3 Centro de Investigación y Asistencia en Tecnología y Diseño del Estado de Jalisco A.C., Guadalajara 44270, Jalisco, Mexico

**Keywords:** proanthocyanidins, gut microbiota, *Akkermansia*, in vitro fermentation, wash-out

## Abstract

**Background**: Phenolic compounds, particularly anthocyanins and proanthocyanidins (PACs), are poorly absorbed in the upper digestive tract and reach the colon largely intact, where they may influence gut microbiota (GM) composition and, in turn, impact host health. We hypothesized that a PAC-rich aronia extract would beneficially modulate the GM, promote the growth of health-associated bacteria, and enhance short-chain fatty acid (SCFA) production across different colon sections, with partial reversion effects after supplementation ends. **Methods**: The Twin-M-SHIME^®^ system was used to simulate the digestion and colonic fermentation in two donors with contrasting microbiota profiles. The experimental design included four phases: stabilization (14 days), control (7 days), treatment with 500 mg/day PAC-rich aronia extract (21 days), and wash-out (10 days). SCFA production was monitored, and changes in microbiome composition were assessed using 16S rRNA gene sequencing. **Results**: PAC-rich aronia extract significantly modulated SCFA levels, increasing butyrate and reducing acetate, with some inter-donor variability. SCFA concentrations tended to return to baseline after the wash-out (WO) period. Metagenomic analysis revealed a decrease in *Collinsella*, *Sutterella*, *Selenomonas*, and *Parabacteroides*—genera linked to low-fiber diets and gut inflammation—while promoting Proteobacteria (e.g., *Escherichia-Shigella*, *Klebsiella*) and butyrate-associated Firmicutes such as *Lactiplantibacillus*. Although some microbial shifts partially reverted during the wash-out (e.g., *Akkermansia*, *Bacteroides*, and *Bifidobacterium*), other changes persisted. **Conclusions**: These findings suggest that PAC-rich aronia extract beneficially modulates GM and SCFA production, but continuous intake may be necessary to maintain these effects over time.

## 1. Introduction

Chokeberry (*aronia melanocarpa* Medik.), a member of the Rosaceae family, originates from North America and Eastern Canada [1]. The fruit of this plant exhibits strong antioxidant and therapeutic properties, owing to its richness in phenolic compounds (neochlorogenic and chlorogenic acids), anthocyanins (particularly cyanidin-3-galactoside and cyanidin-3-arabinoside), proanthocyanidins (PACs), flavanols, flavonols, and ascorbic acid [2,3,4]. Several studies have highlighted its health-promoting benefits, including cardiovascular protection [5,6,7,8,9], reduced oxidative stress [10], and potential anti-cancer effects. [11,12,13]. Additionally, aronia has been shown to improve metabolism [14,15,16], reduce inflammation by inhibiting pro-inflammatory mediators [17], exhibit antimicrobial activity [18], and provide prebiotic-like effects [19,20].

It is well established that the bioavailability of phenolic compounds—such as phenolic acids, anthocyanins, and proanthocyanidins—is generally low to moderate, largely dictated by their chemical structure, degree of conjugation, molecular weight, and site of release along the gastrointestinal tract. While low-molecular-weight phenolic acids like ferulic and caffeic acid may be absorbed in the small intestine, glycosylated compounds such as anthocyanins and larger polymeric structures like PACs typically escape absorption in the upper gut [21] and reach the colon intact, often representing more than 95% of the ingested dose [22,23]. In the case of anthocyanins, only aglycones are absorbed in the stomach, while the majority are degraded in the small intestine into smaller phenolic compounds under neutral pH conditions [24,25]. PACs, particularly those found in aronia with degrees of polymerization (DP) ranging from 10 to 50 [26], are too large to be absorbed in the upper gastrointestinal tract and therefore reach the colon largely intact. Once in the colon, these high-molecular-weight phenolics are subject to microbial metabolism, a process influenced by both the polymer size and the composition of the gut microbiota. This biotransformation leads to the production of low-molecular-weight phenolic acids, which can be absorbed and may contribute to the health benefits associated with PAC consumption [27].

PACs and anthocyanins, two major classes of flavonoids, exert diverse health benefits, largely through their interactions with the gut microbiota [28,29,30]. Despite their limited absorption, these compounds reach the colon, where they act as prebiotics by promoting the selective growth of beneficial microorganisms, thereby supporting a balanced GM [20,31,32,33]. This selective modulation maintains gut ecosystem homeostasis, resulting in elevated SCFA production that lowers intestinal pH and inhibits the proliferation of pathogenic microbes [34]. In addition, PACs contribute to gut health by stimulating Muc2 expression in goblet cells and reinforcing the intestinal barrier. Additionally, they stimulate the secretion of anorectic hormones, such as PYY and GLP-1, regulate gene expression involved in L-cell differentiation [35], reduce inflammatory markers in plasma (TNF-α, IL-6, and MCP-1), decrease epidydimal fat mass, and enhance insulin sensitivity [36]. Anthocyanins complement these effects by affecting the growth of SCFA-producing bacteria, thereby acidifying the gut environment and inhibiting pathogenic bacteria [18,37,38,39]. Furthermore, (poly)phenol-derived metabolites have been shown to selectively promote beneficial microbial taxa while suppressing opportunistic or pathogenic species, thereby reinforcing their role in maintaining gut homeostasis. Taken together, these properties highlight the potential of PACs and anthocyanins to act as “duplibiotics”—a term proposed to describe compounds that combine prebiotic activity with antimicrobial properties, fostering a eubiotic gut environment supportive of host health [40].

We hypothesize that a PAC-rich aronia extract positively modulates the GM through multiple mechanisms, including the stimulation of short-chain fatty acid (SCFA) production, particularly propionate and butyrate, and the selective promotion of beneficial microbiota taxa. Additionally, we aim to assess the impact of a wash-out period on GM resilience and the persistence of the modulatory effect following the withdrawal of the polyphenol supplement. To investigate these effects, we administered a PAC-rich aronia extract to the GM in a Simulator of the Human Intestinal Microbial Ecosystem (Twin-M-SHIME^®^) and evaluated SCFA production and GM modulation using 16S rRNA gene amplicon sequencing.

## 2. Results

### 2.1. Inter-Individual Variability of Donors’ Microbiota and Their Production of SCFAs

**Figure 1:**
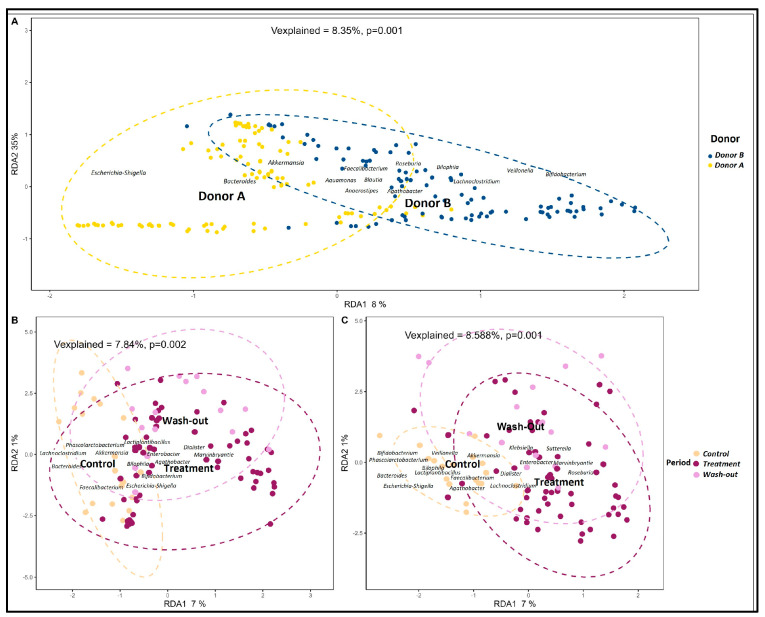
Gut microbiota composition during evaluation in Twin-M-SHIME^®^. Distance-based redundancy analysis (db-RDA) of the GM, determined by 16S rRNA gene amplicon sequencing, highlights the differences between donors (inter-individual variability). RDA1 and RDA2 represent the percentage of total variance explained, while “*Vexplained*” indicates the eccentricity of this variability and its significance, evaluated using a distance matrix PERMANOVA. (**A**) GM composition from both donors: donor A (yellow), and donor B (blue). (**B**) GM distribution across evaluation periods for donor A: Control (beige), Treatment (plum), and WO (pink). (**C**) GM distribution across evaluation to periods for donor B, using the same color scheme as donor A.

The donors exhibited distinct GM profiles. For donor A, the predominant genera were *Escherichia-Shigella*, *Akkermansia*, *Bacteroides*, *Aquamonas*, *Lachnospiraceae*, *Anaeroglobus*, and *Selenomonas*, while donor B was characterized by *Akkermansia*, *Bifidobacterium*, *Bacteroides*, *Veillonella*, *Lachnoclostridium*, *Anaerostipes*, and *Bilophila.* Figure 1A shows that the GM profiles of both donors were significantly different (*p* = 0.001), as determined by a PERMANOVA. Figure 1B,C depict the evolution of GM distribution throughout the Twin-M-SHIME^®^ experiment, highlighting the effects of the supplementation with a PAC-rich aronia extract.

As shown in Table 1, α-diversity and richness (assessed by the Chao index) significantly increased only in the lumen of the transverse colon (TC) during the WO period for donor A, and in the ascending colon (AC) during the treatment period for donor B (*p* < 0.05, Kruskal–Wallis). In contrast, no marked differences were observed between treatments for the Shannon index, except in the AC lumen during the WO period for donor A, in the TC lumen during the treatment period for both donors, and in the TC mucus during the treatment period only for donor A.

**Figure 2:**
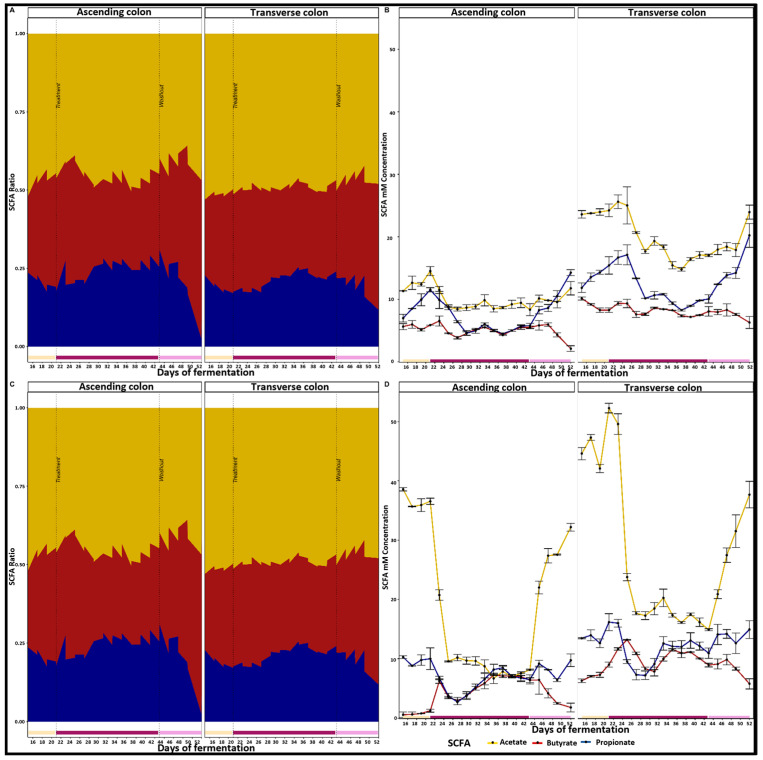
Effect of a 500 mg PAC-rich aronia extract on the SCFAs production in AC and TC. Panels (**A**,**B**) display SCFA ratio and concentrations (in mM) respectively, for donor A across the Control (Days 15–21), Treatment (Days 22–42), and WO (Days 43–52) periods. Similarly, panels (**C**,**D**) show SCFAs ratios and concentrations for donor B over the same evaluation periods in the Twin-M-SHIME^®^ system. Dashed lines in panels (**A**,**C**) mark the onset of the Treatment and WO periods. The yellow, red, and blue zones in panels (**A**,**C**) represent acetate, butyrate, and propionate ratios, respectively. In panels (**B**,**D**), the yellow, red, and blue lines indicate acetate, butyrate, and propionate concentrations in mM.

Given the differences between the microbiomes of the two donors, their metabolic outputs were likewise distinct. Figure 2 illustrates the production of SCFAs throughout the experiment, highlighting the differential responses of the donors’ microbiota: donor B showed a significant increase in butyrate production during the treatment period (*p* = 0.0001 in the AC and *p* = 0.0005 in the TC). While donor A also experienced an increase in butyrate production, this increase was not as high as that observed in donor B in the TC (*p* = 0.0309).

Conversely, propionate production significantly decreased in both donors, but only in the AC (*p* = 0.0004 for donor A, and *p* = 0.0005 for donor B). Both donors also exhibited a notable drop in acetate levels from the treatment period through the WO period (*p* = 0.0002 for AC and *p* < 0.0001 for TC in donor A; *p* < 0.0001 for both colon sections in donor B). By the end of the WO period, SCFA levels in both donors tended to revert to those observed during the Control period.

### 2.2. Effects of PAC-Rich Extract Supplementation on Microbiota Composition

**Figure 3:**
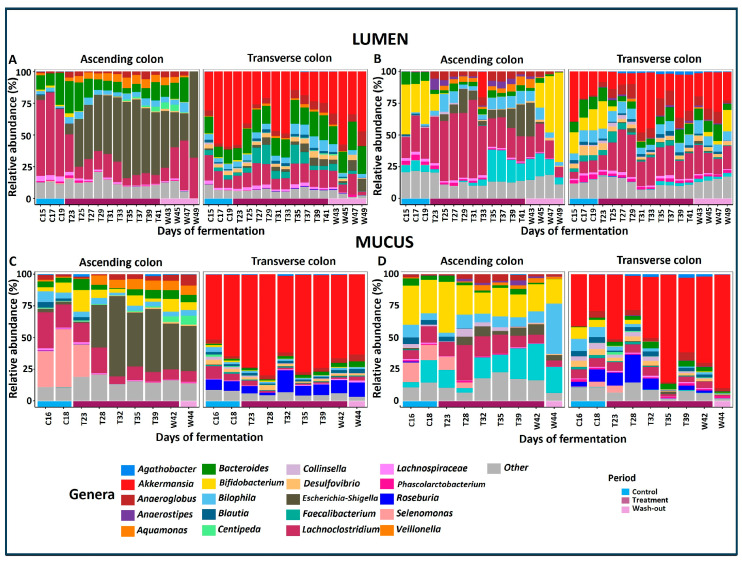
Genus-level relative abundance of gut microbiota from donor samples during a Twin-M-SHIME® fermentation experiment. Figures (**A**,**B**) illustrate GM composition in the lumen for Donors A and B, respectively, while figures (**C**,**D**) show the composition in the mucus for Donors A and B, respectively. The x-axis indicates the evaluation periods: Control (blue line), Treatment (violet line), and WO (lilac line).

As illustrated in Figure 3, both donors and niches exhibited distinct GM profiles across the evaluation periods. In the lumen of donor A’s AC (Figure 3A), the dominant genera were *Lachnoclostridium*, *Bacteroides*, and *Escherichia-Shigella*, which shifted during the treatment period. *Lachnoclostridium* decreased, while *Escherichia-Shigella*, *Bacteroides*, *Anaeroglobus*, and *Aquamonas* increased. These changes partially reverted to the Control period profile during the WO period. In the TC lumen, *Akkermansia* was predominant throughout the treatment period, alongside *Lachnoclostridium*, *Bacteroides*, and *Anaeroglobus*, with increases in *Faecalibacterium* and *Escherichia-Shigella*.

In the mucus of the AC section (Figure 3C), the dominant genus included *Selenomonas*, *Lachnoclostridium*, *Bilophila*, and *Bifidobacterium.* The treatment resulted in increases in *Escherichia-Shigella*, *Aquamonas*, and *Anaeroglobus*, while *Lachnoclostridium* and *Centipeda* decreased; notably, *Centipeda* increased during the WO period. In the mucus of the TC section, *Akkermansia*, remained the dominant genus across all evaluation periods, while *Roseburia* decreased during the first week of treatment but returned to its initial levels during the remaining Treatment and WO periods.

Figure 3B displays the 20 most abundant genera in donor B’s AC and TC lumen throughout the evaluation. In the AC, *Bifidobacterium*, *Lachnoclostridium*, *Bacteroides*, and *Veillonella* dominated during the Control period. Treatment led to an increase in *Lachnoclostridium*, *Anaerostipes*, *Escherichia-Shigella*, and *Anaeroglobus*, with a decrease in *Bifidobacterium*, which tended to recover during the WO period. In the TC lumen, *Akkermansia* dominated alongside *Bifidobacterium* and *Bacteroides* during the Control period. Treatment induced an increase in *Lachnoclostridium*, *Anaeroglobus*, *Bilophila*, *Anaerostipes,* and *Agathobacter*, with a decline in *Bifidobacterium*, *Veillonella,* and *Phascolarctobacterium. Bifidobacterium* partially recovered during the WO period.

Figure 3D illustrates the 20 most abundant genera in the mucus of donor B’s AC and TC sections. In the AC, *Bifidobacterium* was the dominant genus during the Control period, followed by *Selenomonas*, *Bilophila*, *Lachnoclostridium*, *Blautia*, and *Bacteroides*. Treatment led to a decrease in *Bifidobacterium*, *Blautia*, and *Selenomonas*, and an increase in *Lachnoclostridium*, *Escherichia-Shigella*, *Collinsella*, *Anaerostipes*, and *Anaeroglobus*, with *Bilophila* continuing to increase during the WO period. In the TC mucus, *Akkermansia* remained predominant throughout the evaluation, followed by *Bifidobacterium*, *Bilophila*, *Lachnoclostridium*, and *Desulfovibrio* during the Control period. Treatment resulted in a decrease in *Bifidobacterium*, *Desulfovibrio*, *Bilophila*, *Selenomonas*, and *Collinsella*, with *Akkermansia* dominating the GM at the end of the evaluation. A detailed contrast analysis of these results is provided later.

**Figure 4:**
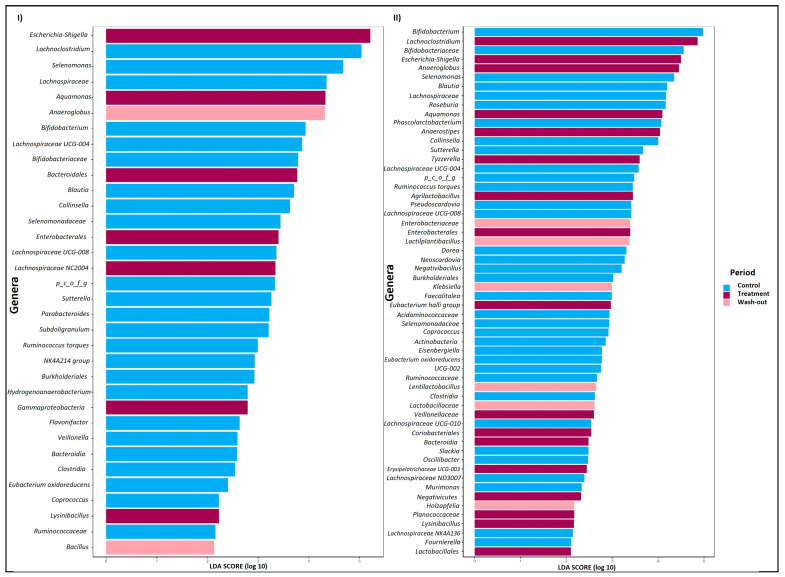
Effects of PAC-rich supplementation on gut microbiota composition. Linear discriminant analysis (LDA) effect size (LEfSe) was calculated to identify the taxa that most strongly discriminated between evaluation periods in Twin-M-SHIME^®^ (Control, Treatment, and Wash-out). Panel (**l**) shows the LEfSe results for donor A, while panel (**II**) shows the results LEfSe for donor B. Grouped data were analyzed using the Kruskal–Wallis test, with a significant set to 0.05, to determine if the data were differentially distributed between groups.

In Figure 4, the Linear discriminant analysis Effect Size (LEfSe) and the corresponding Linear Discriminant Analysis (LDA) scores revealed the predominant microorganisms enriched during each period. During PAC supplementation, there was a bloom of *Escherichia-Shigella* and stimulation of *Aquamonas*, *Lachnospiraceae NC2004*, and *Lysinibacillus* for donor A. In contrast, donor B showed an abundance of genera such as *Lachnoclostridium*, *Escherichia-Shigella*, *Anaeroglobus*, *Aquamonas*, *Anaerostipes*, *Tyzzerella*, *Agrilactobacillus*, *Eubacterium hallii* group, and *Lysinibacillus*. These findings underscore the distinct microbial responses elicited in each donor during the PAC intervention period.

**Figure 5:**
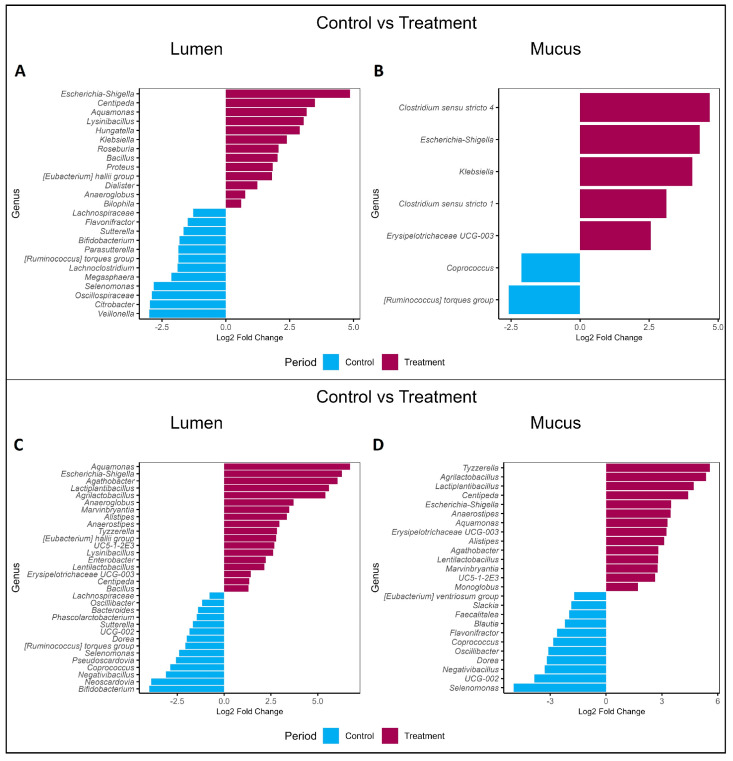
Comparison between the gut microbiome of the control period and after the treatment periods. Figures (**A**,**B**) represent the lumen and mucus microbiota from donor A, while Figures (**C**,**D**) correspond to the lumen and mucus microbiota from donor B.

Supplementation with PAC-rich aronia extract led to significant shifts in the GM composition of both donors, as revealed by the differential expression (DESeq) analysis (Figure 5). Proteobacteria, including *Escherichia-Shigella*, *Hungatella*, *Tyzzerella*, and *Aquamonas*, increased significantly in abundance (*p* < 0.05), while *Collinsella*, *Sutterella*, and *Selenomonas* decreased, as indicated by the Wald test.

**Figure 6:**
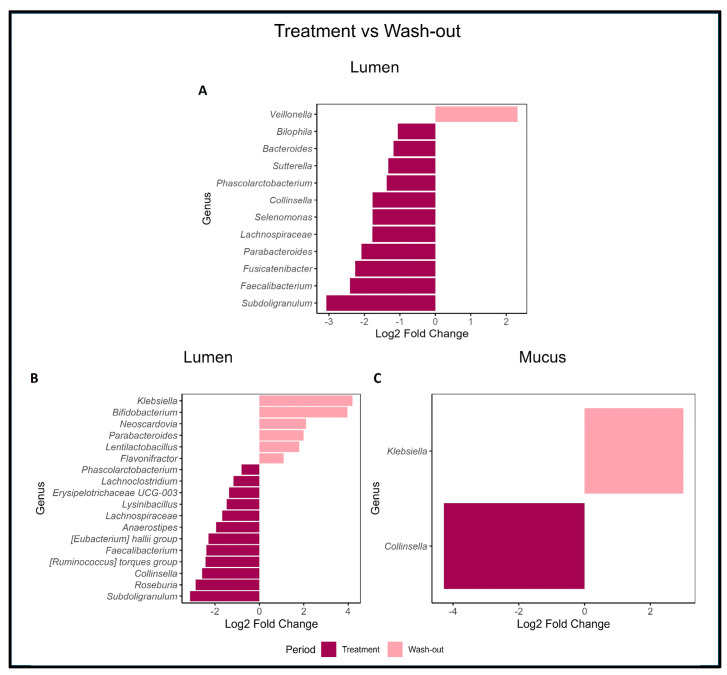
Gut microbiome comparison between Control and Treatment periods. DESeq analysis was conducted to determine differences in GM composition between PAC-rich aronia extract supplementation (Treatment period) and Wash-out period, utilizing the Wald Test with a significance level of α < 0.05 and a Benjamini–Hochberg multiple correction (Stephens, 2016) [41]. Figure (**A**) represents the lumen microbiota from donor A, while Figures (**B**,**C**) correspond to the lumen and mucus microbiota from donor B.

Conversely, the proportion of *Bifidobacterium,* a dominant genus in donor B’s GM, declined in both donors during supplementation with PAC-rich aronia extract, with a particularly significant decrease in donor B (*p* < 0.05), as indicated by the DESeq analysis using Wald test with Benjamini–Hochberg multiple correction. However, at the beginning of the WO period, the proportions of *Bifidobacterium* levels increased again (*p* < 0.05), as shown in Figure 5C and Figure 6D.

Distinct responses were observed between donors. In donor A, supplementation decreased the relative abundance of *Lachnospiraceae*, *Lachnoclostridium*, *Agathobacter*, *Blautia*, *Lysinibacillus*, *Lactiplantibacillus*, and *Citrobacter*, while donor B exhibited decreases in *Blautia*, *Lachnospiraceae*, *Phascolarctobacterium*, *Bacteroides*, *Coprococcus*, and *Dorea*, alongside increases in *Anaeroglobus*, *Lactiplantibacillus*, *Lentilactobacillus*, *Anaerostipes*, and *Tyzzerella.*

Both donors shared similar trends in lumen samples, with increases in *Escherichia-Shigella, Centipeda, Aquamonas, Bacillus*, the *Eubacterium hallii* group, and *Anaeroglobus*, and decreases in *Lachnospiraceae*, *Sutterella*, *Bifidobacterium*, the *Ruminococcus torques* group, and *Selenomonas* during the treatment. In the mucus samples, both donors exhibited increases in *Escherichia*-*Shigella* and *Erysipelotrichaceae UGC-003*, along with a decrease in *Coprococcus*.

Figure 6 presents the DESeq comparison between the Treatment and WO periods in the lumen and mucus niches. In Figure 6A (lumen), donor A exhibited a significant increase (*p* < 0.05) in *Veillonella* during the WO period, alongside a lower abundance in *Bilophila*, *Bacteroides*, *Sutterella*, *Phascolarctobacterium*, *Collinsella*, *Selenomonas*, *Lachnospiraceae*, *Parabacteroides*, *Fusicatenibacter*, *Faecalibacterium*, and *Subdoligranulum* during the Treatment period. In contrast, donor B (Figure 6A) displayed a significant increase in *Klebsiella*, *Bifidobacterium*, *Neoscardovia*, *Parabacteroides*, *Lentilactobacillus*, *Flavonifractor* during the WO period, coupled with lower abundances in *Phascolarctobacterium*, *Lachnoclostridium*, *Erysipelotrichaceae* UCG-003, *Lysinibacillus*, *Lachnospiraceae*, *Anaerostipes*, the *Eubacterium hallii* group, *Faecalibacterium*, the *Ruminococcus torques* group, *Collinsella*, *Roseburia*, and *Subdoligranulum* during the treatment period.

In mucus, no significant differences between the treatment and WO periods in donor A were observed. Conversely, donor B (Figure 6C) exhibited a significant increase in *Klebsiella* during the WO period and a lower relative abundance of *Collinsella* during the treatment period.

Both donors demonstrated similar trends in lumen samples, with decreases in *Phascolarctobacterium*, *Collinsella*, *Lachnospiraceae*, *Faecalibacterium*, and *Subdoligranulum* when comparing the treatment and WO periods.

**Figure 7:**
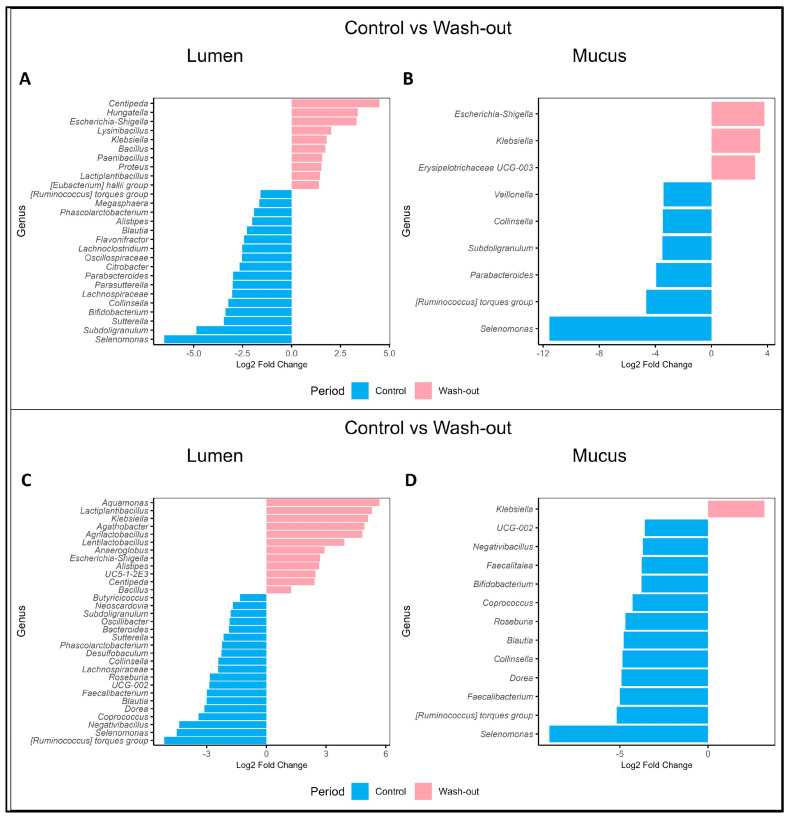
Gut microbiome comparison between Control and Treatment periods. DESeq analysis was conducted to determine differences in GM composition between Control and Wash-out periods, utilizing the Wald Test with a significant level of α < 0.05 and a Benjamini–Hochberg multiple correction. Figures (**A**,**B**) represent the lumen and mucus microbiota from donor A, while Figures (**C**,**D**) correspond to the lumen and mucus microbiota from donor B.

Figure 7 illustrates the DESeq comparison between the Control and WO periods in both lumen and mucus niches. Figure 7A shows that in the lumen of donor A, there was a significant increase (*p* < 0.05) during the WO period in *Centipeda*, *Hungatella*, *Escherichia-Shigella*, *Lysinibacillus*, *Klebsiella*, *Bacillus*, *Paenibacillus*, *Proteus*, *Lactiplantibacillus*, and the *Eubacterium hallii* group. Meanwhile, the relative abundance of the *Ruminococcus torques* group, *Megasphaera*, *Phascolarctobacterium*, *Alistipes*, *Blautia*, *Flavonifractor*, *Lachnoclostridium*, *Oscillospiraceae*, *Citrobacter*, *Parabacteroides*, *Parasutterella*, *Lachnospiraceae*, *Collinsella*, *Bifidobacterium*, *Sutterella*, *Subdoligranulum*, and *Selenomonas* was significantly lower during the Control period.

In donor B, Figure 7C displays a significant increase in *Aquamonas, Lactiplantibacillus, Klebsiella, Agathobacter, Lentilactobacillus, Aneroglobus, Escherichia-Shigella, Alistipes, UC5-1-2E3, Centipeda,* and *Bacillus* during the WO period. Conversely, there was a notable decrease in *Butyricicoccus, Neoscardovia, Subdoligranulum, Oscillibacter, Bacteroides, Sutterella, Phascolarctobacterium, Desulfobaculum, Collinsella, Lachnospiraceae, Roseburia, UCG-002, Faecalibacterium, Blautia, Dorea*, *Coprococcus*, *Negativibacillus*, *Selenomonas*, and the *Ruminococcus torques* group during the Control period.

In the mucus samples, Figure 7B shows that donor A had a significant increases (*p* > 0.05) in *Escherichia-Shigella* and *Klebsiella* during the WO period, while the Control period exhibited lower abundances of *Veillonella*, *Collinsella*, *Subdoligranulum*, *Parabacteroides*, the *Ruminococcus torques* group, and *Selenomonas.* Similarly, in donor B, Figure 7D highlights an increase in *Klebsiella* during the WO period, alongside significant decreases in UCG-002, *Negativibacillus*, *Faecalitalea*, *Bifidobacterium*, *Coprococcus*, *Roseburia*, *Blautia*, *Collinsella*, *Dorea*, *Faecalibacterium*, the *Ruminococcus torques* group, and *Selenomonas* during the Control period.

Both donors demonstrated similar trends in lumen samples, with increases in *Centipeda*, *Escherichia-Shigella*, *Klebsiella*, *Bacillus*, and *Lactiplantibacillus*, during the WO period, and lower abundances in the *Ruminococcus torques* group, *Phascolarctobacterium*, *Blautia*, *Lachnospiraceae*, *Collinsella*, *Sutterella*, and *Subdoligranulum* compared to the Control period. In mucus, both donors exhibited an increase in *Klebsiella* during the WO period, with lower abundances of *Collinsella*, the *Ruminococcus torques* group, and *Selenomonas* during the Control period.

### 2.3. Supplementation with PAC-Rich Aronia Extract Maintains a High Relative Abundance of Akkermansia in Both Donors in the Transverse Colon Tested in Twin-M-SHIME^®^

Both donors’ GM exhibited a notably high relative abundance of the genus *Akkermansia* (Figure 3). Given the unexpected nature of this result, we conducted a more detailed analysis to verify its validity. Interestingly, DESeq analysis indicated no significant differences in *Akkermansia* abundance between the treatment and the control across the three evaluation periods (Control, Treatment, and WO), suggesting that its levels were not treatment-responsive but rather dependent on donor-specific factors. Although bar plots in Figure 3 show *Akkermansia* dominance in the mucus of both donors, statistical analysis confirms this abundance was consistent across periods.

**Figure 8:**
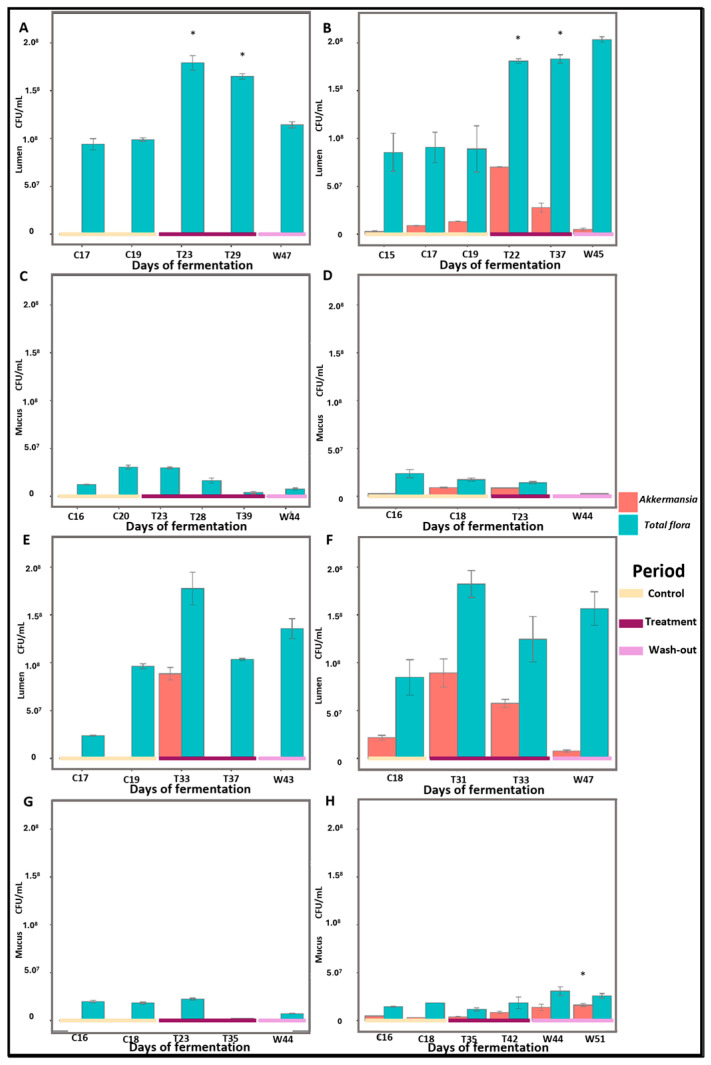
Quantification (CFU/mL) of total flora (blue bars) vs. *Akkermansia* (orange bars) during Twin-M-SHIME^®^ evaluation. Bar plots representing the quantification of total flora and *Akkermansia* for both donors were evaluated separately. Figures (**A**–**D**) correspond to donor A’s samples from the lumen AC, lumen TC, mucus AC, and mucus TC, respectively. Figures (**E**–**H**) correspond to donor B’s samples from the same compartments (lumen AC, lumen TC, mucus AC, and mucus TC, respectively). The *x*-axis shows the evaluation periods: Control period (beige line), Treatment period (violet line), and WO period (lilac line). Statistical significance is indicated by * (*p* < 0.05).

Interestingly, one donor displayed an unexpectedly high proportion of *Akkermansia* in the Twin-M-SHIME^®^ samples. Given the unexpected nature of this result, we conducted a more detailed analysis to verify its validity. This finding was validated by ddqPCR (Figure 8), which confirmed the relative abundance to CFU/mL, ensuring that the ratio remained consistent when analyzed using qPCR. This validation ruled out the possibility of bias due to a suspected antimicrobial effect.

The results from the one-way ANOVA for each target (*p* > 0.05) indicated an increase in total bacterial counts when comparing the Control and Treatment periods, observed only in the lumen of donor A, along with a significant increase in *Akkermansia* in the TC compartment in the mucus of donor B. However, neither the bacterial populations nor *Akkermansia* (CFU/mL) exhibited significant changes following supplementation with the PAC-rich aronia extract. In summary, these findings were consistent with the 16S sequencing results across the Twin-M-SHIME^®^ evaluation.

### 2.4. Contrasting Recovery: A Comparative Analysis of Control and Wash-Out Periods

As for SCFAs, several genera commonly found in the colon, including *Akkermansia*, *Bacteroides*, *Bifidobacterium*, *Escherichia-Shigella*, *Lachnoclostridium*, and *Veillonella*, exhibited a tendency to revert to their original abundance levels after the supplementation in PAC-rich aronia extract was discontinued. Genera that did not significantly differ between the Control and the WO periods were further analyzed using a qualitative similarity test via Venn diagrams.

**Figure 9:**
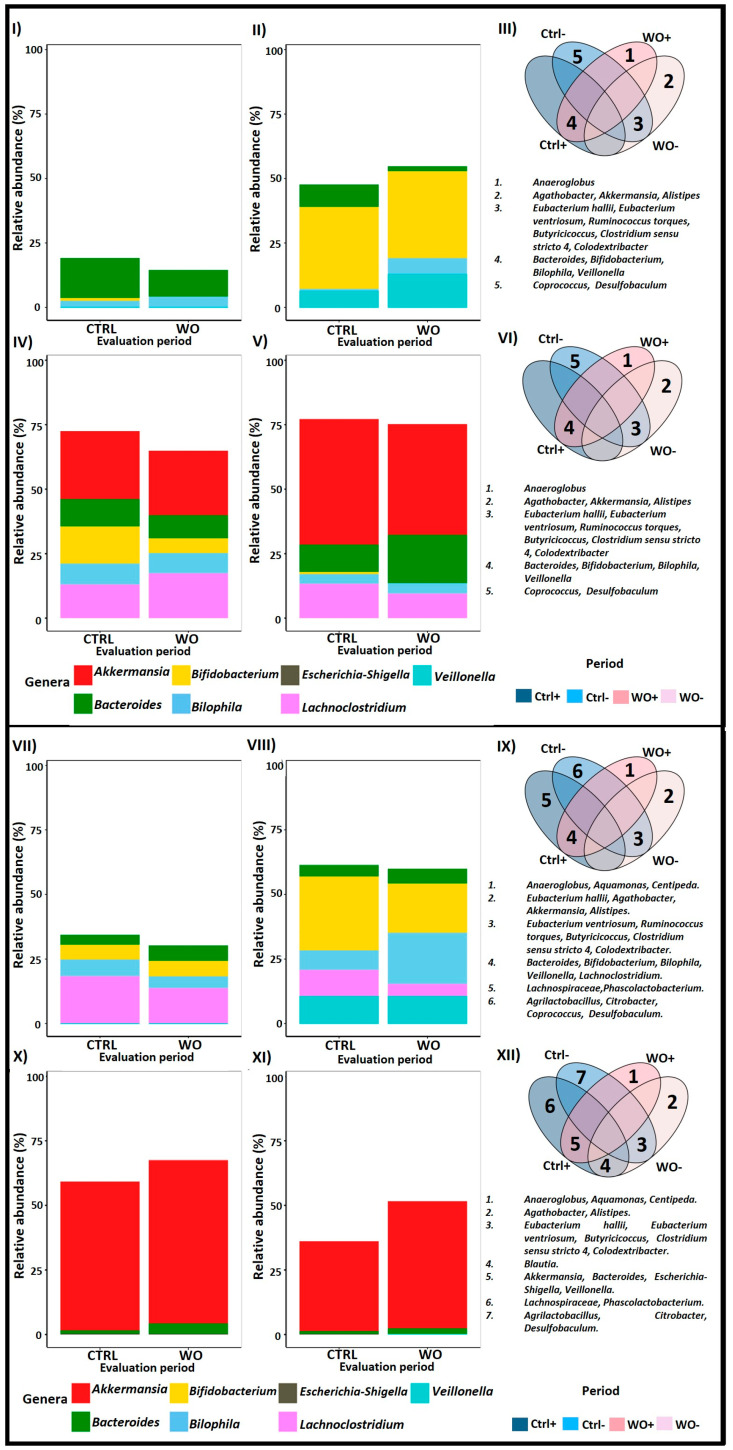
Similarity test between Control and Wash-out period. Venn diagrams display the overlap of the most abundant (CTRL+ and WO+) and less abundant (CTRL− and WO−) genera in different colon sections from Twin-M-SHIME^®^. Bar plots show the non-significant relative abundance of the genus common to both periods. Panels (**I**–**III**) represent the lumen of the ascending colon for donors A and B, respectively. Panels (**IV**–**VI**) represent the lumen of the transverse colon for donors A and B, respectively. Panels (**VII**–**IX**) represent the mucus of the ascending colon for donors A and B, respectively. Panels (**X**–**XII**) represent the mucus of the transverse colon for donors A and B, respectively.

Panels I to III in Figure 9 highlight that *Bacteroides*, *Bifidobacterium*, *Biophilia*, and *Veillonella* remained among the most abundant genera during both the Control and WO periods in the lumen of both donors in AC. Conversely, genera such as *Coprococcus*, *Desulfobaculum*, the *Eubacterium hallii* group, the *Eubacterium ventriosum* group, the *Ruminococcus torques* group, *Butyricicoccus*, *Clostridium sensu stricto 4*, and *Colidexitribacter* consistently remained at low abundance across the same periods in the lumen.

Similarly, panels IV to VI illustrate that *Akkermansia*, *Bacteroides*, *Bifidobacterium*, *Bilophila*, and *Lachnoclostridium* were among the most abundant genera during the Control and WO periods in the lumen of both donors in TC. In contrast, genera such as the *Eubacterium hallii* group, the *Eubacterium ventriosum* group, the *Ruminococcus torques* group, *Butyricicoccus*, *Clostridium sensu stricto 4*, and *Colidexitribacter* consistently maintained low abundance.

Panels VII to IX reveal that *Bacteroides*, *Bifidobacterium*, *Bilophila*, *Lachnoclostridium*, and *Veillonella* remained among the most abundant genera during the Control and WO periods in the mucus of both donors in AC. Conversely, genera like the *Eubacterium ventriosum* group, the *Ruminococcus torques* group, *Butyricicoccus*, *Clostridium sensu stricto 4*, and *Colidexitribacter* consistently maintained lower abundance in the mucus.

Lastly, panels X to XIII show that *Akkermansia* and *Bacteroides* were among the most abundant genera during both the Control and WO periods in the mucus of both donors in TC. In contrast, genera such as the *Eubacterium hallii* group, the *Eubacterium ventriosum* group, *Butyricicoccus*, *Clostridium sensu stricto 4*, and *Colidexitribacter* consistently maintained low abundance in the mucus.

## 3. Discussion

Considering the expanding evidence highlighting the critical role of GM in host health [42,43,44], this research aimed to explore the interactions between aronia (poly)phenols with GM. This focus is particularly relevant for PAC, as they are minimally absorbed in the upper digestive tract and reach the colon intact—especially those with a DP > 4 [45,46,47,48,49,50], such as those evaluated in this study, since aronia PAC have an exceptionally high DP (~29), as is shown in Materials and Methods. The interaction between PAC and GM is considered bidirectional: the properties of PAC influence the composition of GM, while only specific microorganisms possess the capacity to metabolize PAC [51,52,53]. However, this relationship is not yet fully understood and may reveal key mechanisms underlying their health-promoting effects.

To enhance our understanding of how supplementing 500 mg of aronia PAC-rich extract impacted the GM, we conducted an in vitro evaluation that consisted of a 7-day Control period, 21-day treatment with the extract, followed by a 10-day WO period, using the Twin-M-SHIME^®^. This system was inoculated with fecal samples from two healthy individuals. The Twin-M-SHIME^®^ effectively mimics the physiology of the gastrointestinal tract, capturing its dynamic nature. Moreover, it enables the simulation of both GM niches: lumen, and mucus.

Daily supplementation with a proanthocyanidin-rich aronia extract modulated SCFA production by reducing acetate levels and enhancing butyrate concentrations. Our findings show a significant decrease in colonic acetate levels, accompanied by a marked increase in butyrate production, suggesting that the PAC-rich aronia extract may have stimulated the activity or growth of butyrate-producing bacteria. This shift in the SCFA profile aligns with the observations of Wu et al. (2018) [4], who reported a reduction in acetate following aronia juice supplementation in a SHIME^®^ model. In their study, *Bifidobacterium* was positively correlated with acetate, while members of the Firmicutes phylum were negatively correlated with acetate and positively associated with butyrate levels [4]. Similarly, our findings are supported by studies on cranberry PAC-rich extract supplementation, which also reported comparable effects in SCFA modulation [31]. In our study, the observed decrease in *Bifidobacterium* abundance may explain the reduction in acetate production, while the increased levels of several Firmicutes genera, including *Aquamonas*, *Lysinibacillus*, *Lactiplantibacillus, Anaeroglobus*, *Bacillus*, *Lentilactobacillus*, *Anaerostipes*, and *Tyzzerella*, could be related to the increased butyrate synthesis. These results suggest that PAC-rich aronia extract supplementation may beneficially influence GM metabolic activity by promoting butyrate production while reducing acetate, potentially contributing to improving gut health.

The PAC-rich aronia extract promoted an increase in Proteobacteria, including *Escherichia-Shigella*, *Centipeda*, *Aquamonas*, *Proteus*, *Bilophila*, *Klebsiella*, and *Citrobacter.* This increase could be attributed to the high concentration of (poly)phenols in the extract, which likely exerted selective influence on the GM by inhibiting the growth of certain Gram-positive bacteria, such as Firmicutes and Bacteroidetes, thereby allowing more resistant Gram-negative Proteobacteria to proliferate [54,55]. A comparative study by Taguri et al. (2004) demonstrated that (poly)phenols displayed stronger antibacterial activity against Gram-positive bacteria, including *Staphylococcus aureus*, while showing lower efficacy against Gram-negative bacteria like *Escherichia coli* [56]. Nevertheless, Ivanov et al. (2022) reported that some (poly)phenols can also inhibit antibiotic-resistant bacteria, including certain Gram-negative strains [57]. Similarly, Wu et al. (2018) [4] found that aronia juice stimulated the growth of Proteobacteria, such as *Sutterella*, *Aeromonas*, and Enterobacteriaceae family. Increases in Proteobacteria have also been observed in response to PAC-rich cranberry extract in the Twin-M-SHIME^®^ model by Cattero et al. (2024) [31], and following administration of a high dose of blueberry (poly)phenols (1000 mg/Kg/day) in vivo by Cladis et al. (2021) [58]. Likewise, Mayta-Apaza et al. (2018) reported similar findings after supplementation with tart cherry (poly)phenols, both in vitro and in vivo [59]. Together, these observations suggest that supplementation with PAC-rich extracts may selectively modulate GM by promoting the growth of certain Proteobacteria, likely due to their greater resistance to (poly)phenols compared to other microbial groups.

An increase in specific Firmicutes genera was observed following supplementation with the PAC-rich aronia extract, suggesting their potential role in (poly)phenol metabolism. Our analysis revealed an elevated abundance of *Aquamonas*, *Lysinibacillus*, *Lactiplantibacillus, Anaeroglobus*, *Bacillus*, *Lentilactobacillus*, *Anaerostipes*, and *Tyzzerella*. This trend is consistent with findings by Wu et al. (2018), who reported an increase in *Lachnospiraceae*, *Anaerostipes*, *Megasphaera*, *Lactobacillus*, and members of Ruminococcaceae family following aronia juice supplementation [4]. These observations reinforce the idea that PAC-rich aronia extracts can modulate the GM by promoting the growth of specific bacterial taxa potentially involved in the metabolism of complex polyphenols, highlighting the influence of dietary polyphenols on microbial community structure.

We anticipated that PAC-rich aronia extract supplementation would stimulate *Akkermansia*, as numerous studies have reported similar effects following the administration of PAC-rich fruits in both in vitro and in vivo models. However, supplementation did not result in significant changes in either the relative abundance or CFU/mL of *Akkermansia;* instead, its levels remained stable throughout the intervention. This stability is particularly noteworthy, considering that the baseline GM already exhibited a high abundance of *Akkermansia*. A possible mechanism for the resilience of this genus, despite the potential antimicrobial effects of (poly)phenols, may be the one proposed by Rodríguez-Daza et al. (2021) [40]. They suggested that *Akkermansia*’s tolerance to phenolic compounds, together with reduced competition from opportunistic microorganisms suppressed by the antimicrobial activity of (poly)phenols, may enable this bacterium to sustain its proliferation. In the present study, *Akkermansia* did not proliferate but remained stable in the presence of the PAC-rich extract, possibly benefiting from the decline of competing microorganisms [40]. These findings differ from those of Wu et al. (2018), who reported that aronia juice treatment stimulated the proliferation of *Akkermansia* [4]. Altogether, this suggests that while PAC-rich treatments can promote *Akkermansia* in some contexts, they may also support its stability in communities where it is already abundant, despite broader shifts in the GM of both donors.

Our results revealed a decrease in the relative abundance of the *Bifidobacterium* genus during the treatment. This observation aligns with previous findings by Wu et al. (2018) following aronia juice supplementation [4], and by Kemperman et al. (2013) using black tea and red wine/grape juice supplementation [60]. Notably, all three studies were conducted using the SHIME^®^ system. In contrast, other studies on (poly)phenols supplementation from different sources reported an increase in the relative abundance of *Bifidobacterium*. For example, Cattero et al. (2024) [31] found that a PAC-rich cranberry extract supplementation in Twin-M-SHIME^®^ promotes *Bifidobacterium* proliferation. However, this extract had a significantly lower DP (DP = 8) [31,52]. Similarly, Bialonska et al. (2010) observed an increase in *Bifidobacterium* abundance following pomegranate by-product supplementation in a fecal batch fermentation model, although their study focused on smaller molecules, specifically punicalagins [61]. Likewise, Lessard-Lord et al. (2023) found that *Bifidobacterium* was favored when testing epicatechin in batch fecal fermentation [51]. Furthermore, Whitman et al. evaluated a blend of (poly)phenols (cranberry, blueberry, green tea, and cocoa with an undefined DP) and a fiber blend (resistant starch, galacto-oligosaccharides, and inulin) in an in vitro fermentation model. They found that both the (poly)phenol and fiber blends, as well as the combination, significantly increased *Bifidobacterium* proliferation [62]. Additionally, Van den Abbele et al. (2021) demonstrated that *Bifidobacterium* has a preference for low-DP inulin [63], and other studies suggest that small molecules may facilitate its rapid metabolism [64,65]. These findings suggest that the response of *Bifidobacterium* to (poly)phenol supplementation may depend on multiple factors, including the molecular size, chemical composition, source of the (poly)phenols, and the presence of polysaccharides, as this genus appears to favor the metabolism of smaller molecules, which are more accessible.

Interestingly, supplementation with PAC-rich aronia extract led to a decrease in certain bacteria associated with gut inflammation, such as *Sutterella.* This genus has been linked to various human diseases, including inflammatory bowel disease and autism. Although *Sutterella* has been associated with dysbiotic microbiota, its direct role in the etiopathology of these conditions remains unclear, and it is generally considered a commensal microorganism [66]. Similarly, Li et al. (2020) [67] demonstrated that resveratrol supplementation in mice significantly reduced the relative abundance of *Sutterella*, *Dorea*, and *Bilophila* while also attenuating colitis. Additionally, they found that those genera were positively correlated with pro-inflammatory cytokines, including IL-1β, IL-6, and IFN- γ [67]. These findings support the idea that (poly)phenol-rich interventions, such as those with aronia, may help to reduce gut inflammation by modulating microbial populations associated with pro-inflammatory cytokines.

Our results demonstrate that PAC-rich aronia extract significantly decreases the relative abundance of *Collinsella*. This genus has been positively associated with low fiber intake and a reduced expression of tight junction proteins in enterocytes—factors linked to conditions such as leaky gut and endotoxemia [68]. Moreover, *Collinsella* abundance has shown a positive correlation with circulating insulin levels in overweight and obese pregnant women, with higher levels noted in obese participants. These findings suggest that the reduction in *Collinsella* induced by PAC-rich aronia extract may contribute to improving gut barrier integrity and metabolic health in the context of dietary interventions.

Donors exhibited distinct microbial responses to supplementation with the PAC-rich aronia extract. In the lumen, the treatment increased the abundance of *Hungatella*, *Roseburia*, *Proteus*, *Dialister*, and *Bilophila*, while reducing *Flavonifractor*, *Parasutterella*, *Lachnoclostridium*, and *Megasphaera* in donor A. In contrast, donor B showed an increase in *Agathobacter*, *Lactiplantibacillus*, *Agrilactobacillus*, *Marvinbryantia*, *Alistipes*, *Anaerostipes*, *Tyzzerella*, *UC5-1*, *Enterobacter*, *Lentilactobacillus*, and *Erysipelotrichaceae UCG-003*, alongside a decrease in *Oscillibacter*, *Phascolarctobacterium*, *UCG-002*, *Dorea*, *Pseudoscardovia*, *Coprococcus*, *Negativibacillus*, *Neoscardovia*, and *Bifidobacterium.* In mucus, donor A exhibited an increase in *Clostridium sensu stricto 4* and *1*, as well as *Klebsiella*, while *Ruminococcus torques* decreased. Donor B, on the other hand, displayed a rise in *Tyzzerella*, *Agrilactobacillus*, *Lactiplantibacillus*, *Centipeda*, *Anaerostipes*, *Aquamonas*, *Alistipes*, *Agathobacter*, *Lentilactobacillus*, *Marvinbryantia*, *UC5-1-2E3*, and *Monoglobus*, with reductions in *Eubacterium ventriosum*, *Slackia*, *Faecalitalea*, *Blautia*, *Flavonifractor*, *Oscillibacter*, *Dorea*, *Negativibacillus*, *UCG-002*, and *Selenomonas.* These changes were not uniform; some genera increased only in specific colon sections or varied between donors. A randomized, placebo-controlled parallel clinical trial conducted by Lackner et al. (2024) [20] assessed the effects of consuming 400 mL/day of aronia juice, containing 8.33 g/L of total (poly)phenols, over a 6-week period followed by a 6-week WO period, reporting an increase in *Anaerostipes* and *Bacteroides* after supplementation, with a decline during the WO period. These findings partially align with our results, although the study did not specify the composition or DP of the (poly)phenols used [19,20]. Additionally, the authors suggested that these two genera might play a role in the tolerability of aronia juice, potentially contributing to inter-individual variability in gut microbiota modulation. Our findings highlight the inter-individual variability in GM responses to PAC-rich aronia extract supplementation, with distinct shifts in microbial composition across donors and intestinal regions.

Donor B exhibited an increase in *Anaerostipes* during the PAC-rich aronia extract supplementation. An increase in *Anaerostipes*, a butyrate producer via the butyryl-CoA: acetate CoA-transferase pathway [69], has been previously reported concerning aronia (poly)phenols, particularly as a result of cross-feeding in the butyrate production during aronia juice supplementation containing 6.5 g of total (poly)phenols [4]. Furthermore, an increase in both *Anaerostipes* and *Bacteroides* was observed in a double-blind randomized controlled trial involving 12 weeks of supplementation with a (poly)phenol extract that contained only 16 mg of PAC and a total of 116 mg of (poly)phenols [6]. Additionally, an increase in *Bacteroides* was noted with an extract of aronia that was particularly rich in anthocyanins [70]. These findings suggest that the increase in *Anaerostipes* observed in donor B may be linked to the metabolism of SCFA, potentially through cross-feeding mechanisms that contribute to butyrate production.

Both donors displayed high abundances in *Akkermansia* and Bacteriores and exhibited similar microbial trends in response to treatment. Supplementation with the PAC-rich aronia extract led to an increase in *Escherichia-Shigella*, *Centipeda*, *Aquamonas*, *Bacillus*, the *Eubacterium hallii* group, and *Anaeroglobus*, while reducing *Lachnospiraceae*, *Sutterella*, *Bifidobacterium*, the *Ruminococcus torques* group, and *Selenomonas* in the luminal compartment of both donors. In the mucus niche, both donors showed an increase in *Escherichia-Shigella* and *Erysipelotrichaceae* UGC-003, along with a decrease in *Coprococcus*. Additionally, as previously mentioned, acetate levels declined while butyrate production increased for both donors. These findings suggest that, although individual GM profiles differ, treatment with a specific compound can elicit similar microbial responses across distinct communities. Notably, Holmes, et al. (2022) demonstrated that despite interindividual variability, the GM responded in a conserved manner to certain prebiotics, indicating that specific dietary interventions can lead to consistent functional outcomes across diverse GM compositions [71]. Other studies have shown that GM can influence the efficacy of therapies, such as cancer treatments, with specific microbial functions correlating with positive responses in different individuals, despite differences in taxonomic composition [72]. Together, these findings suggest that while microbial community structures vary among individuals, their functional potential, particularly regarding SCFA production, may converge in response to targeted interventions, enabling comparable physiological outcomes.

The limited availability of studies evaluating the biological effects of purified high-DP PAC presents a challenge in comparing our results. Our extract had a notably high DP of approximately 29, which is relatively rare among published studies. Our findings suggest that such highly polymerized PACs may have limited bioavailability and microbial metabolism. This aligns with the work of Mena et al. (2018), who noted that PAC metabolism remains controversial, particularly with high molecular weight and A-type PACs, which appear more resistant to microbial catabolism [73]. Supporting this, Lessard-Lord et al. (2024) concluded that aronia polymeric B-type PACs and cranberry oligomeric A-type PAC were not metabolizable by the GM [52]. Ichikawa et al. (2022) used the highest DP PAC compound reported to date (DP = 14.33) to investigate effects on human T-lymphotropic retrovirus-infected adult T-cell leukemia cells [74], but they observed no significant results for the high-DP PAC fraction. Similarly, Andersen-Civil et al. (2021) tested different PAC polymers in mouse macrophage models and found that high-DP PACs (DP = 12.3) showed less biological activity compared to lower-DP PACs [75]. These findings collectively suggest that the bioactivity of PACs may be inversely related to their polymer size, with smaller molecules being more effective in modulating host and microbial responses.

Our research included a 10-day WO period aimed at evaluating whether the effects of the treatment were sustained after supplementation cessation. We observed that only seven GM genera and SCFA levels tended to return to baseline, indicating partial reversion. This duration is slightly longer than most studies, where, WO periods commonly range from two days to one week [76,77]. For example, Koper et al. (2022) documented a one-week WO period after tryptophan supplementation observing similar partial reversions [77]. In contrast, Van den Abbeele et al. (2013) employed a two-week WO period in a SHIME^®^ model and found persistent effects with long-chain arabinoxylan supplementation but not with inulin [78]. These observations highlight that the persistence of treatment effects varies depending on the supplementation type and WO duration. Altogether, these insights underscore the importance of considering both the compound supplemented and the duration of WO periods when evaluating long-term microbiota modulation.

The reversion to baseline levels might suggest that, although the treatment significantly altered the GM composition during the intervention, the overall microbiome structure retains its capacity to restore pre-treatment SCFA levels over time, as acetate levels increased again, while butyrate levels decreased to concentrations similar to the control period. Several studies have highlighted the remarkable resilience of gut microbiota to external perturbations. Fragiadakis et al. (2020) showed that, although a three-month dietary intervention induced significant alterations in gut microbiota composition, the community largely reverted toward its baseline structure despite continued dietary adherence and sustained weight loss. Similarly, short-term antibiotic interventions were reported to cause only transient perturbations, with both the gut microbiota and its resistome rapidly recovering baseline configurations within a week [79]. These findings emphasize that, even when microbial community composition is not fully restored, functional outputs such as SCFA production tend to recover over time. This suggests that the gut microbiota may exhibit functional redundancy, allowing it to maintain essential metabolic activities, such as SCFA synthesis, even after significant compositional shifts induced by dietary or other interventions.

Our results further underscore the variability in microbiota responses to supplementation, with each donor exhibiting a distinct shift in bacterial composition. This variability may be influenced by baseline microbiota composition, environmental factors, and genetic predisposition, all of which contribute to the complexity of microbial dynamics. Despite these differences, our findings provide insights into the stability of certain microbial populations, such as *Akkermansia*, *Bacteroides*, and *Bilophila* in general, which remained consistently abundant throughout the evaluation. This stability suggests a potential role in maintaining gut homeostasis, both in microbial composition and overall functionality (SCFA production). To broaden the relevance of our findings, future studies should include a larger and more diverse group of donors. This would provide a deeper understanding of interindividual variability and help determine how consistently the gut microbiota responds to dietary interventions across different microbial ecosystems. Such data are essential for identifying the key factors that influence microbiota stability and responsiveness, ultimately supporting the translation of these results to human populations.

The PAC-rich aronia extract evaluated in this study induced significant shifts in gut microbiota composition and enhanced butyrate production—both widely regarded as hallmarks of prebiotic-like activity. These findings suggest that the extract may modulate the gut environment in ways that support host health. However, as outlined by the International Scientific Association for Probiotics and Prebiotics (ISAPP), classification as a prebiotic requires more stringent criteria: the compound must possess a well-defined chemical structure and composition, be selectively utilized by host microorganisms, and demonstrate consistent microbiota modulation associated with a measurable health benefit. Furthermore, a mechanistic rationale should underpin how the microbial changes contribute to the observed benefit, and the compound’s safety must be established at the effective dose [80,81,82]. While (poly)phenols such as PAC and anthocyanins reach the colon largely unabsorbed and undergo microbial biotransformation, their classification as prebiotics necessitates clear evidence of selective utilization by specific microbial taxa and demonstrable health benefits [81]. Although the aronia extract used in this study is rich in PAC and also contains phenolic acids, anthocyanins, and polysaccharides, further investigation is needed to pinpoint the specific compounds driving the observed effects. Therefore, while our findings support prebiotic-like activity, definitive classification of the extract as a prebiotic will require mechanistic studies confirming selective microbial utilization and associated physiological benefits.

## 4. Materials and Methods

### 4.1. PAC-Rich Aronia Extract

The composition of a PAC-rich aronia extract, supplied by Symrise (Diana Food Canada Inc., Champlain, QC, Canada), was described previously [40] (Supplementary Table S1). Apart from its high content of chlorogenic, anthocyanins, and phenolic acids, it is especially rich in high-molecular-weight PAC, with an average DP of about 29.

### 4.2. Reagents

All reagents used for the Twin-M-SHIME^®^ evaluation and sample processing are described in the Supplementary Materials.

### 4.3. Twin-M-SHIME^®^

**Figure 10:**
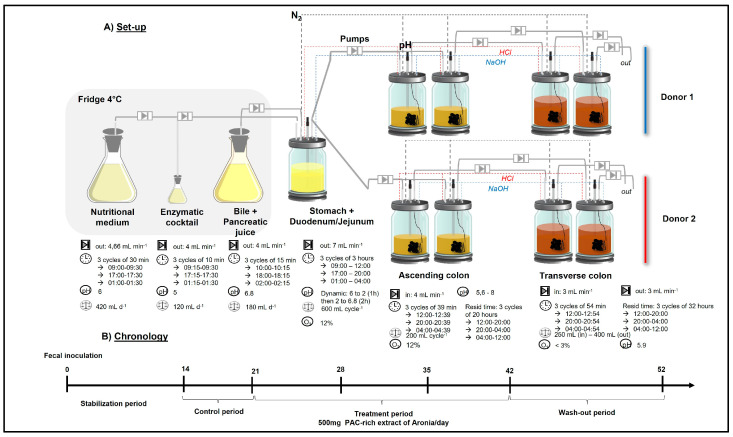
Twin-M-SHIME^®^ experimental design. (**A**) Diagram of the Twin-M-SHIME^®^ fermentation system, featuring reactors in series from the stomach/small intestine (SI) through to the ascending and transverse colon. The colonic reactors for each unit were inoculated with samples from two different fecal donors, with each donor replicated in duplicates. (**B**) Timeline showing the three experimental periods and their durations. The experiment lasted 52 days, beginning with a 14-day stabilization period, followed by a 7-day control period. The treatment period lasted 21 days, during which 500 mg of PAC-rich aronia extract was added daily to the SHIME^®^ stomach, progressing through catabolism in the ascending and transverse colon. Finally, a 10-day WO period followed. The sampling schedule is detailed in the Supplementary Materials.

The Twin-M-SHIME^®^ system was used to conduct the current study to simulate human intestinal microbiota from lumen and mucus. This system replicates the microbial environments of the human large intestine. In this study’s setup, the first vessel simulates the stomach (ST) and small intestine (SI) using a fill-and-draw method, where a nutritional medium is introduced along with pancreatic and bile juices. This is followed by two vessels representing the AC and TC, acknowledging that PAC reach the colon intact, where their oligomers and dimers begin their degradation by GM in the AC. Both the AC and the CT are maintained at fixed volumes. The detailed operation of the system has been previously described by Cattero et al. (2024) [31], illustrated in Figure 10. Briefly, the fecal inoculum used to seed the Twin-M-SHIME^®^ system was prepared from two healthy donors by mixing fresh fecal matter with anaerobic phosphate buffer at 20% (*w*/*v*) concentration, following a previously established method (Van den Abbeele et al., 2021 [63]). The bioreactors were inoculated with 5% (*w*/*v*) of this fecal solution. During fermentation, microbiotas were fed three times daily with a standard SHIME^®^ nutritional medium, containing floating mucin and bile acids. To simulate mucus-associated microbiotas in the ascending and transverse colons, microcosms (AnoxKaldnes K1 carrier) coated with type II porcine mucin-agar (MUC2 gel-forming mucin) were employed.

Samples from the colon vessels were collected every two days, centrifuged, and stored at −20 °C for subsequent SCFAs and DNA analysis. Additionally, mucus-associated microbiota samples from the microcosms were collected three times per week for analysis. Mucus aliquots from the colon vessels were obtained using a mini sampler spoon and stored at −20 °C for DNA extraction.

### 4.4. Short-Chain Fatty Acid Analysis

Daily 125 µL samples were collected at 12:00 from each compartment of the Twin-M-SHIME^®^ system. The fecal water was centrifuged at 14,000 rpm for 8 min at 4 °C, after which the supernatant was collected and stored at −80 °C until further processing. Before the analysis, 154 selected samples were randomized, numbered, and prepared for SCFA quantification via gas chromatography, following Roussel. et al. (2022) [38].

### 4.5. Metagenomics Analysis

The samples collected during the Twin-M-SHIME^®^ experiment were processed for DNA isolation and purification following the protocol reported by Cattero et al. (2024) [31]. The DNA samples with a concentration >10 ng/µL were subjected to next-generation sequencing of the 16S rRNA gene amplicons, specifically targeting the V3–V4 region (341F-805R). Sequencing was carried out at the Institut de Biologie Intégrative et des Systèmes (IBIS) at Université Laval, Canada, utilizing an Illumina MiSeq platform with V3 chemistry and a 600-cycle reagent kit (Illumina, San Diego, CA, USA).

Amplicon sequence variants (ASVs) were identified using the Denoising Algorithm (DADA2) workflow, implemented in the dada2 R package (version 1.8) [83]. This software package allows for error modeling and correction in Illumina-sequenced amplicons [83]. The taxonomic assignation was performed using the SILVA database [84].

### 4.6. Absolute Quantification of PCR Targets for Total Bacteria and Akkermansia muciniphila

To assess whether the relative abundance of *Akkermansia muciniphila* accurately reflected its actual quantity in the microbiota—given a suspected overall decrease in all microbial genera except *Akkermansia*, which could bias relative abundance measurements—a quantitative PCR (qPCR) analysis was conducted. Genomic DNA samples from the Twin-M-SHIME^®^ were subjected to droplet digital PCR (ddPCR) using primers for *A. muciniphila* (forward 5′-CACACCGCCCGTCACAT-3′ and reverse 5′-TGCGGTTGGCTTCAGATACTT-3′) [85]. The total bacterial count was estimated by using the universal primers Uni334F (5′-ACTCCTACGGGAGGCAGCAGT-3′) and Uni514R (5′-ATTACCGCGGCTGCTGGC-3) [86].

The assays were performed at IBIS, using a QX 200 ddPCR System, and the data were analyzed using QuantaSoft™ Analysis Pro 1.0.596 (Bio-Rad, Hercules, CA, USA). Once the standard curve for each target was generated, previously sequenced genomic DNA samples from all evaluation periods (Control, Treatment, and WO), all colon sections (AC and TC), and from both donors were selected to calculate colony-forming units per milliliter (CFU/mL) via qPCR.

### 4.7. Statistical Analysis

Statistical analysis and figure generation for SCFAs and metagenomics data were performed using R (version 4.3.2). Microbial community α-diversity during the evaluation was calculated using Chao and Shannon indices, and the Krustal–Wallis test was applied to compare means. These analyses were conducted using the Vegan R package (version 2.6–4) and ggpubr package (version 0.6.0). Additionally, distance-based redundancy analysis (db-RDA) was conducted to compare donor microbiomes, niches, colon sections, and the effects of treatment during the Twin-M-SHIME^®^ evaluation. A permutational analysis of variance (PERMANOVA) was applied to determine the significance of group separation.

The DESeq2 package (version 1.42.0) was used to identify significant differences in species abundance across evaluation periods (Control, Treatment, and Wash-out). The results were visualized with bar plots, boxplots, and volcano plots, with *p*-values calculated using the Benjamini–Hochberg post hoc test, as described by [31].

Finally, LDA and LEfSe were applied to identify microbial taxa that significantly differed between evaluation periods. A threshold LDA score of >2.0 was set, as per [87], to distinguish these taxa during the Twin-M-SHIME^®^ evaluation.

## 5. Conclusions

21-day administration of 500 mg of PAC-rich aronia extract modulated the gut microbiome by significantly promoting the increase in Proteobacteria, particularly from the Enterobacteriaceae family, as well as Firmicutes, such as *Aquamonas*, *Lysinibacillus*, *Lactiplantibacillus, Anaeroglobus*, *Bacillus*, *Lentilactobacillus*, *Anaerostipes*, and *Tyzzerella*, which may be associated with (poly)phenol metabolism. This intervention also resulted in a marked decrease in acetate production, while slightly enhancing butyrate production in two contrasting donors. After cessation of the treatment, SCFA levels returned to baseline, whereas the gut microbiota composition remained substantially altered. Only selected genera, such as *Akkermansia* (which maintained stable levels throughout the study), *Bacteroides*, *Bifidobacterium, Escherichia-Shigella, Bilophila, Lachnoclostridium*, and *Veillonella*, exhibited a partial reversion toward their control-period abundances. These findings suggest that regular intake of the (poly)phenols extracts is necessary to maintain stable alterations in GM composition and SCFA levels induced by supplementation.

## Figures and Tables

**Table 1:** Effect of PAC-rich aronia extract supplementation on the microbiota’s richness and diversity.

GM	Donor	Colon	Period	Chao Index (10^4^)	Shannon Index	Simpson Index
Lumen	A	AC	Control	18.22 ± 7.28	3.97 ± 0.33	0.85 ± 0.03
			Treatment	18.33 ± 9.20	4.12 ± 0.21	0.88 ± 0.03
			Wash-out	50.06 ± 8.10	5.07 ± 1.26 ***	0.93 ± 0.03
		TC	Control	9.86 ± 3.89	4.74 ± 0.44	0.90 ± 0.04
			Treatment	9.25 ± 3.39	5.26 ± 0.38 *	0.95 ± 0.03
			Wash-out	78.31 ± 10.50 **	5.70 ± 1.52	0.93 ± 0.07
	B	AC	Control	18.11 ± 4.71	4.64 ± 0.27	0.93 ± 0.02
			Treatment	13.40 ± 6.31 *	4.79 ± 0.40	0.94 ± 0.03
			Wash-out	20.21 ± 13.46	4.66 ± 0.37	0.93 ± 0.03
		TC	Control	16.84 ± 12.01	5.15 ± 0.24	0.96 ± 0.01
			Treatment	9.13 ± 2.88	5.47 ± 0.36*	0.96 ± 0.03
			Wash-out	25.77 ± 3.89	5.42 ± 1.05	0.94 ± 0.06
Mucus	A	AC	Control	17.61 ± 5.12	4.48 ± 0.56	0.92 ± 0.03
			Treatment	35.57 ± 57.08	4.40 ± 1.11	0.89 ± 0.06
			Wash-out	23.97 ± 21.04	4.60 ± 0.28	0.93 ± 0.02
		TC	Control	39.03 ± 12.48	4.26 ± 0.32	0.86 ± 0.04
			Treatment	36.61 ± 42.12	3.41 ± 0.57 **	0.73 ± 0.10
			Wash-out	119.03 ± 13.33	5.30 ± 2.89	0.85 ± 0.20
	B	AC	Control	15.92 ± 10.83	4.44 ± 0.77	0.92 ± 0.06
			Treatment	38.15 ± 6.08	4.95 ± 0.89	0.95 ± 0.02
			Wash-out	115.83 ± 14.07	6.29 ± 1.90	0.98 ± 0.01
		TC	Control	22.72 ± 0.56	4.92 ± 0.16	0.95 ± 0.01
			Treatment	49.93 ± 7.75	4.73 ± 1.63	0.85 ± 0.15
			Wash-out	119.99 ± 13.51	5.24 ± 3.36	0.81 ± 0.26

Comparisons were made with respect to the control. Significance levels are indicated as follows: * *p* < 0.05; ** *p* < 0.01; *** *p* < 0.001.

## Data Availability

The original data presented in this study are openly available at: https://www.ncbi.nlm.nih.gov/bioproject/PRJNA1267035 (accessed on 29 April 2025).

## Supplementary Materials

The following supporting information can be downloaded at: https://www.mdpi.com/article/10.3390/ph18060793/s1, Table S1: (Poly)phenol composition of Aronia extract by UPLC-UV-QToF; Table S2. Nutritional SHIME^®^ medium composition; Table S3. Pancreatic juice composition; Table S4. Twin-M-SHIME^®^ setup; Table S5. Sampling schedule during the complete 52-days evaluation in the Twin-M-SHIME^®^; and Figure S1. Distance-based redundancy analysis (db-RDA) of gut microbiota, determined by 16S rRNA gene amplicon sequencing, highlights the differences between donors (inter-individual variability).

## Abbreviations

The following abbreviations are used in this manuscript:

PAC	Proanthocyanidins
GM	Gut microbiota
SCFA	Short-chain fatty acids
Twin-M-SHIME^®^	Twin Mucosal Simulator of the Human Intestinal Microbial Ecosystem
rRNA	Ribosomal ribonucleic acid
WO	Wash-out
DP	Degree of polymerization
Muc2	Mucin 2
PYY	Peptide tyrosine tyrosine
GLP-1	Glucagon-like peptide-1
TNF-α	Tumor necrosis factor alpha
IL	Interleukin
MCP-1	SCFAs
PERMANOVA	Permutational multivariate analysis of variance
AC	Ascending colon
TC	Descending colon
db-RDA	Distance-based redundancy analysis
RDA	Redundancy analysis
mM	Millimolar
LEfSe	Linear discriminant analysis effect size
LDA	Linear discriminant analysis
DESeq	Differential gene expression analysis of RNA-seq data
ddPCR	Droplet digital polymerase chain reaction
CFU	Colony-forming unit
mL	Milliliter
ANOVA	Analysis of variance
ST	Stomach
SI	Small intestine
v/w	Volume/weight
DNA	Deoxynucleic acid
min	Minutes
°C	Degrees Celsius
µl	Microliter
ng	Nanogram
mg	Milligram
IBIS	Institut de Biologie Intégrative et des Systèmes
ASVs	Amplicon sequence variants
qPCR	Quantitative polymerase chain reaction
