# Supplementary material for: Prebiotic-like Effects of Proanthocyanidin-Rich Aronia Extract Supplementation on Gut Microbiota Composition and Function in the Twin-M-SHIME® Model
Source: Pharmaceuticals (Basel). 2025 May 25;18(6):793. doi: 10.3390/ph18060793 (PMC12196067; doi:10.3390/ph18060793)
Supplement: Supplementary file 1 [file pharmaceuticals-18-00793-s001.zip › pharmaceuticals-3643195-supplementary.pdf]

Supplementary Materials

Table S1. (Poly)phenol composition of Aronia extract by UPLC-UV-QToF.

	Concentration (mg/g) (Mean ± Standard Deviation)
Flavan-3-ols monomers total	1.39 ± 0.04
Procyanidin B2	2.21 ± 0.010
Phenolic acids total	114.6 ± 0.5
Flavonols (total)	18.17 ± 0.05
Anthocyanins (total)	49.7 ± 1.3
PAC (total)	219.5 ± 0.2
Polysaccharides (total)	101.0 ± 2.0
PAC Mean DP	29 ± 0.6

Twin-M-SHIME® description

The first vessel from the Twin-M-SHIME® system, i. e., the stomach, was filled three times daily with 140 mL of nutritional medium at a pH of 2, with a 45-minute residence time to simulate stomach physiological conditions. The composition of the nutritional medium is provided in the table below:

Table S2. Nutritional SHIME® medium composition

	**g/L**
Arabinogalactan (TCI)	1.2
Pectin (Acros Organics)	2.0
Xylan (TCI)	0.5
Glucose (Alfa Aesar)	0.4
Yeast extract (BioBasic)	3.0
Proteose peptone (Oxoid)	1.0
Mucin (Carl Roth by ProDigest)	2.0
L-cystein HCl (BioBasic)	0.5
Starch (Fisher)	4.0

Following the 45-minute residence period after nutritional medium delivery, 60 mL of pancreatic and bile juices were introduced, allowing for a 1 hour and 30-minute residence time at a pH of 6.8 to replicate the transition into the small intestine. The composition of the pancreatic juice is provided in the table below.

Table S3. Pancreatic juice composition

	**g/L**
NaHCO3 (Fisher)	12.5
Bovine Bile salts (Difco 212820)	6.0
Pancreatin 4Xusp	0.9

Following this, the gradual transfer between vessels occurred over 40 minutes. The ascending colon vessels were filled with 250 mL of fecal slurry, maintaining a pH range of 5.6-5.8 and a hydraulic residence time of 20 hours. The transverse colon vessels held 500 mL of fecal slurry, with the pH adjusted to a range of 6.25-6.4 and a hydraulic residence time of 32 hours. Further details are provided in the table below.

Table S4. Twin-M-SHIME® setup

Compartment	Volume per unit	pH	Mucin beads	Pumps	Pump type	1 st cycle	2nd cycle	3rd cycle
NUTRITIONAL MEDIUM X1	140 mL cycle-1 (420 mL d-1)	2	-	Out 4.66 mL/min-1	Fast1 X1	℗ 9:00-9:30	℗ 17:00-17:30	℗ 1:00-1:30
PANCREATIC JUICE/BILE X1	60 mL cycle-1 (180 mL d-1)	6.8	-	Out 4 mL/min-1	Fast1 X1	℗ 10:15-10:30	℗ 18:15-18:30	℗ 2:15-2:30
STOMACH/ SMALL INTESTINE X2	200 mL cycle-1 (600 mL d-1)	Dynamic 6 to 2 (1h) then 2 to 6.8 (2h)	-	Residence time	-	9:00 – 12:00	17:00 – 20:00	1:00– 4:00
				Out 4 mL/ min-1	Slow2 X2	℗12 - 12:39	℗20:00 - 20:39	℗4- 4:39
ASCENDING COLON (AC) X2	250 mL	5.6 – 8	2 x 15	Hydraulic residence time		20 h	20 h	20 h
				Out 3 mL/min-1	Slow2 X2	℗12:00 -12:54	℗20:00 – 20:54	℗4:00 – 4:54
TRANSVERSE COLON (TC) X2	400 mL	6.25 – 6.4	2 x 15	Hydraulic residence time		32 h	32 h	32 h
				Out 3 mL/min-1	Slow2 X2	℗12:00 -12:59	℗20:00 – 20:59	℗4:00 – 4:59

Table S5. Sampling schedule during the complete 52-days evaluation in the Twin-M-SHIME®

	Day	Lumen DNA	Mucin DNA	SCFAs
Stabilization	0	X		X
	3	X	X	X
	6	X	X	X
	9	X	X	X
	12	X	X	X
	13	X	X	X
	14	X	X	X
Control	15	X		X
	16		X	
	17	X		X
	18		X	
	19	X		X
	20			
	21	X		X
Treatmen	22	X		X
	23	X	X	X
	24			

	25	X		X
	26			
	27	X		X
	28		X	
	29	X		X
	30			
	31	X		X
	32		X	
	33	X		X
	34			
	35	X	X	X
	36			
	37	X		X
	38			
	39	X	X	X
	40			
	41	X		X
	42		X	
Wash-out	43	X		X
	44		X	
	45	X		X
	46			
	47	X	X	X
	48			
	49	X	X	X
	50			
	51	X	X	X
	52	X		X

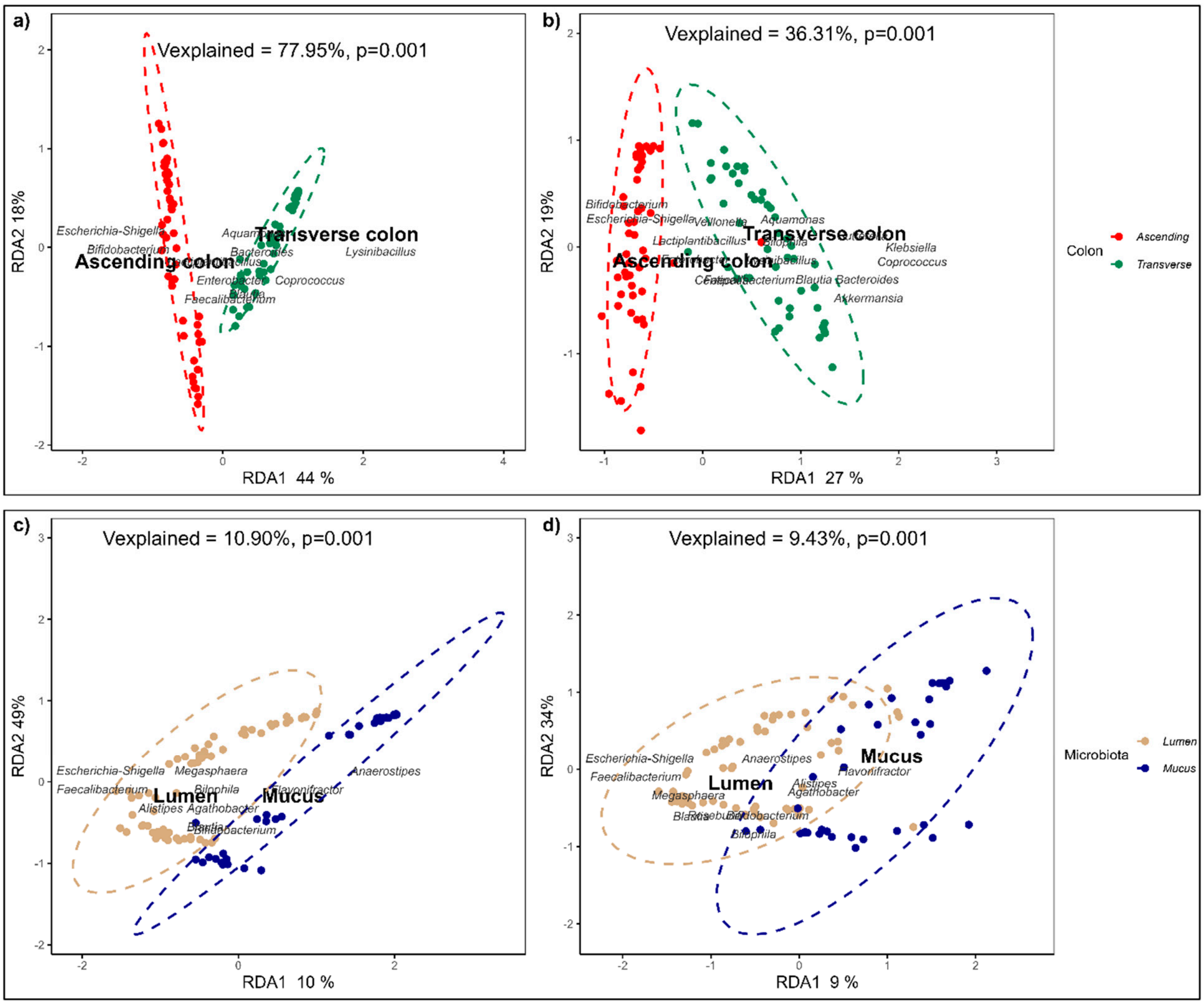

Figure S1. Distance-based redundancy analysis (db-RDA) of gut microbiota, determined by 16S rRNA gene amplicon sequencing, highlights the differences between donors (inter-individual variability). RDA1 and RDA2 represent the percentage of total variance, whilst "*Vexplained*" indicating the variability, assessed for significance through PERMANOVA on the distance matrix. Figures a) and b) show GM composition in both colon sections, AC (red) and TC (green) for donor A and B, respectively. Figures c) and d) illustrate the GM composition in both Lumen (almond) and Mucus (blue) microbiota for donor A and B, respectively.
